# Anti-citrullinated protein antibodies are associated with decreased bone mineral density: baseline data from a register of early arthritis patients

**DOI:** 10.1007/s00296-017-3674-9

**Published:** 2017-02-27

**Authors:** Irene Llorente, Leticia Merino, Ana M. Ortiz, Eugenio Escolano, Saturnino González-Ortega, Rosario García-Vicuña, Jesús A. García-Vadillo, Santos Castañeda, Isidoro González-Álvaro

**Affiliations:** 1 Rheumatology Department, Hospital Universitario de La Princesa, IIS-Princesa, C/ Diego de León 62, 28006 Madrid, Spain; 2 Radiology Department, Hospital Universitario de La Princesa, IIS-Princesa, Madrid, Spain; 3 Rheumatology Department, Hospital San Pedro, Logroño, Spain

**Keywords:** Rheumatoid arthritis, Bone mineral density, Autoantibodies, Autoimmunity

## Abstract

Since the previous studies showed that anti-citrullinated protein antibodies (ACPA) can induce osteoclasts differentiation and activation, even before arthritis onset, the aim of our study was to determine whether ACPA-positivity is associated with lower bone mineral density (BMD) at baseline visit of a register of early arthritis (EA) patients. The study population comprised 578 patients (80% females) from our EA clinic with a median disease duration, 5.1 months (p25–p75: 6–8); median age, 53.6 years (41.9–66.1), 38% ACPA-positive, and 55% fulfilling 2010 criteria for rheumatoid arthritis. BMD was measured using dual X-ray absorptiometry at lumbar spine, hip, and metacarpophalangeal (MCP) joints of the non-dominant hand to evaluate both systemic and juxta-articular bone mass. ACPA titers were determined through enzyme immunoassay. The effect of ACPA on BMD was analyzed using multivariable analysis based on generalized linear models adjusted for various confounders. ACPA-positive patients showed lower bone mass at lumbar spine and hip, but no differences were observed at MCP joints compared to ACPA-negative patients. However, ACPA-positive patients displayed higher disease activity and disability than ACPA-negative patients. After adjustment for gender, age, body mass index, and other bone-related variables, the presence of ACPA remained significantly associated with lower BMD at the lumbar spine, femoral neck, and hip but not at MCP joints. Disease activity was not associated with baseline bone mass. Our data reinforce the previous preclinical findings suggesting that the systemic bone loss detected at the initial phases of early ACPA-positive arthritis is independent of inflammatory status and, therefore, could be mediated by ACPA.

**Electronic supplementary material:**

The online version of this article (doi:10.1007/s00296-017-3674-9) contains supplementary material, which is available to authorized users.

## Introduction

Rheumatoid arthritis (RA) is a chronic inflammatory disease characterized by persistent inflammation of the synovial membrane and joint destruction, bone loss, and systemic complications. Skeletal changes in RA include juxta-articular bone erosions, periarticular bone loss, and systemic osteoporosis [1, 2]. Until a few years ago, rheumatologists assumed that osteoporosis in RA was mainly derived from chronic inflammation, use of glucocorticoids or some disease-modifying anti-rheumatic drugs (DMARDs), and immobilization. However, in the light of current knowledge, bone destruction in arthritis seems to be caused by two main mechanisms: inflammation and autoimmunity [2, 3].

Pro-inflammatory cytokines such as TNF-α, IL-1, IL-6, and IL-8 enhance the proliferation and differentiation of the monocyte-macrophage lineage, increasing the population of mature osteoclasts [2, 4, 5]. Indeed, the existence of an inflammatory microenvironment from the earliest stages of RA had been proposed to be responsible for the appearance of bone erosions and systemic osteoporosis in these phases of the disease [1, 6].

On the other hand, the presence of rheumatoid factor (RF) and, especially, anti-citrullinated protein autoantibodies (ACPA) is another important risk factor for the development of bone erosions and osteoporosis in RA [2, 3, 7]. In this regard, ACPA can be detected up to 5–10 years before clinical synovitis develops and, especially those with anti-citrullinated vimentin and enolase specificities, have been described to induce formation and activation of osteoclasts in vitro and in a mouse model [5, 8]. In fact, Kleyer et al. have recently demonstrated a decrease in cortical bone mass in a limited population of healthy ACPA-positive subjects without any joint symptom [9].

These interesting data suggest that the presence of ACPA could partially explain the bone loss detected in the initial phases of chronic inflammatory arthritis. Thus, in this work, we analyzed whether the presence of ACPA is associated with differences in bone mineral density (BMD) at hip and lumbar spine to assess systemic bone density and at metacarpophalangeal (MCP) joints to measure juxta-articular bone mass in patients referred to our Early Arthritis Clinic.

## Methods

### Patients

A cross-sectional study was performed in 578 patients with suspected early arthritis submitted to the Princesa Early Arthritis Register Longitudinal (PEARL) study, which started in 2001 and in which data are recorded by protocol at five structured visits (baseline, 6, 12, 24, and 60 months). The information registered includes, age, gender, race, disease duration at the beginning of follow-up, smoking status, menopause, family history of RA; therapies received and cumulative prednisone dose at recruitment; global disease activity on a 100-mm visual analogue scale assessed by both the patient and the physician; number of swollen and tender joints (28-joint count), and the score of the Spanish version of the Health Assessment Questionnaire [10]. Laboratory tests include blood cell counts, general biochemistry, erythrocyte sedimentation rate (ESR), C-reactive protein (CRP), rheumatoid factor (RF; measured by nephelometry, positive >20 IU/ml), and anti-citrullinated peptide antibodies (ACPA; assessed by enzyme immunoassay, see below). For this study, disease activity was estimated using the 28-joint disease activity score (DAS28) calculated with the ESR [11] and the Hospital Universitario de La Princesa Index (HUPI) [12], that is an index for the assessment of disease activity in chronic polyarthritis that includes the same domains as DAS28 and SDAI but corrected by gender when considering tender joint count and erythrosedimentation rate (ESR). HUPI is calculated as the sum of four variables (graded 0–3): 28 tender and swollen joint counts, global disease assessment by physician and acute phase reactants. The score of these variables was based in their quartile distribution in the population used to describe this index [12, 13]. A more detailed description of the PEARL study has been previously published [14].

For this work, we used only information from the baseline visit of patients included in the register from February 2002, when we included BMD measurements in the register protocol, until January 2016.

### BMD measurements

BMD was assessed using dual-energy X-ray absorptiometry (DXA) on a Hologic©QDR-4500 Elite (Bedford, MA, USA) at lumbar spine (LS) and hip. Furthermore, in 2004, we started to scan BMD at non-dominant hand to study the effect of joint swelling on juxta-articular bone mass.

Specifically, we analyzed BMD from L2 to L4, total hip (TH) and femoral neck (FN), and at hand, we assessed BMD from second to fifth MCP joints, as previously described [15]. BMD is expressed in g/cm^2^, except for the *β* coefficients in the multivariable analysis that are expressed in mg/cm^2^ to obtain more affordable values.

### ACPA and anti–mutated citrullinated vimentin antibodies

ACPA were measured using a second-generation anti-citrullinated cyclic peptide enzyme immunoassay (EIA; Euro-Diagnostica Immunoscan RA; positive >50 U/ml) until October 2010 and then using a third-generation EIA (QUANTA Lite CCP3 IgG and IgA, Inova Diagnostics; positive >40 U/ml). Both methods are EIA, but the third-generation analysis is able to detect IgA ACPA in addition to IgG antibodies, with no other important differences between them. For this study, ACPA levels were classified as negative if below the manufacturer’s limit, low if above this limit but below the median of the positive population (500 U/ml for the Euro-diagnostica kit and 350 U/ml for the Quanta Lite Kit) and high when above the median of the positive population.

In addition, we assessed anti-mutated citrullinated vimentin IgG antibodies (MCV-ACPA) through a quantitative EIA (ORG548 anti-MCV, Orgentec Diagnostika GmbH, Mainz, Germany; positive >20 U/ml). MCV-ACPA levels were also clustered as defined above for ACPA.

### Ethical statements

PEARL study is conducted according to the principles expressed in the Helsinki Declaration of 1983 and it was approved by the Research Ethics Committee of Hospital Universitario La Princesa. All patients signed a written consent at study entry.

### Statistical analysis

The descriptive analysis was performed by calculating the mean and standard deviation (SD) of quantitative variables with a normal distribution. The median and the interquartile range (IQR) were calculated for those variables with no normal distribution. Estimation of the proportions was used to describe qualitative variables. Student’s *t* test was applied to compare the means of variables with a normal distribution and Mann–Whitney test used for variables that did not present normal distribution. The *χ* ^2^ test was used for qualitative variables.

We first used the t test to determine whether the differences in BMD at the different anatomic sites between ACPA-positive and ACPA-negative patients were statistically significant. However, since there were significant differences between ACPA-positive and ACPA-negative populations in variables that can influence BMD (Table 1), we performed a multivariable analysis through generalized linear models using the glm command of Stata 12.1 for Windows (Stata Corp LP, College Station, TX, USA) for each location. Variables that were different between the two populations (Table 1) as well as those considered relevant to explain BMD (age, body mass index [BMI], smoking, disease activity, and cumulative prednisone dose at baseline) were included in the initial models. The final models were obtained through manual stepwise backward elimination of variables by means of the Bayesian information criterion, removing all variables with *p* > 0.15. The only exception was ACPA status, which was maintained in all the models, even though it did not reach a *p* ≤ 0.15. We also performed a sensitivity analysis by repeating the multivariable analysis both in the population fulfilling the 2010 RA criteria and in patients who did not meet these criteria separately [16].

**Table 1:** Characteristics of the population

	Total (*n* = 578)	ACPA+ (*n* = 220)	ACPA− (*n* = 358)	*p*
Female, *n* (%)	458 (79.2)	187 (85.0)	271 (75.7)	0.007
Age (years; p50 [IQR])	53.6 [41.9–66.3]	52.5 [42.0–64.0]	54.1 [41.8–67.3]	0.409
Smoking, *n* (%)				
Never Ever Current	322 (55.7) 127 (22.0) 129 (22.3)	113 (51.4) 52 (23.6) 55 (25.0)	209 (58.3) 75 (21.0) 74 (20.7)	0.272
BMI (p50 [IQR])	26.0 [23.0–29.1]	25.0 [22.5–28.9]	26.4 [23.5–29.3]	0.005
Menopause (%) no/yes/NA	60.1/37.6/2.3	58.1/38.8 /3.1	61.2/36.9/1.9	0.548
Prednisone use, *n* (%)	130 (22.5)	56 (25.4)	74 (20.7)	0.193
Cumulative prednisone dose (mg; p50 [IQR]; mean ± S)	0 [0–125] 168 ± 445	0 [0–125] 159 ± 436	0 [0–125] 174 ± 451	0.749
Disease duration (months; p50 [IQR])	5.0 [2.8–8.2]	5.5 [3.1–8.8]	4.7 [2.5–7.8]	0.017
2010 RA criteria, *n* (%)	316 (54.7)	196 (89.1)	120 (33.5)	<0.001
RF, *n* (%)	254 (43.9)	171 (77.7)	83 (23.2)	<0.001
DAS28(p50 [IQR])	4.1 [3.2–5.4]	4.3 [3.3–5.6]	4.1 [3.1–5.1]	0.054
HUPI	6.5 [9–4]	7 [10–4]	6 [9–4]	0.0230
HAQ(p50 [IQR])	0.875 [1.5–0.375]	0.875 [1.625–0.375]	0.875 [1.5–0.375]	0.795
Swollen MCP (2nd to 4th; p50 [IQR])	0 [0–1]	1 [0–2]	0 [0–1]	0.004

*n* number, *IQR* interquartile range, *ACPA* anti-citrullinated protein antibodies, *p50* 50th percentile or median, *SD* standard deviation, *BMI* body mass index, *NA* not available, *RA* rheumatoid arthritis, *RF* rheumatoid factor, *DAS28* disease activity score based on a 28-joint count, *HUPI* Hospital Universitario La Princesa Index, *HAQ* Health Assessment Questionnaire, *MCP* metacarpophalangeal joints

Significance was set to *p* < 0.0125 due to multiple comparisons in the bivariate analysis and to *p* < 0.05 in the multivariable analysis, since the latter approach ensures enough adjustment to avoid associations by chance.

## Results

### Differences between ACPA-positive and ACPA-negative patients

More ACPA-positive than ACPA-negative patients fulfilled the 2010 RA criteria [16] (Table 1). Patients not fulfilling these criteria suffered from undifferentiated arthritis (UA, 65.8%), spondyloarthropathies (8.9%), osteoarthritis (8.5%), connective tissue disorders (4.3%), and miscellaneous conditions (e.g., gout or viral arthritis) (22.5%). RF positivity, female gender, longer disease duration, lower BMI, and swollen MCP joints were also significantly more frequent in ACPA-positive patients (Table 1). In addition, this population showed a higher disease activity that reached statistical significance with HUPI and was almost significant when estimated by DAS28 (Table 1). Differences in the percentage of patients treated with glucocorticoids and the cumulative prednisone dose used were not significant (Table 1).

### ACPA-positive patients show lower systemic BMD than ACPA-negative

**Fig. 1:**
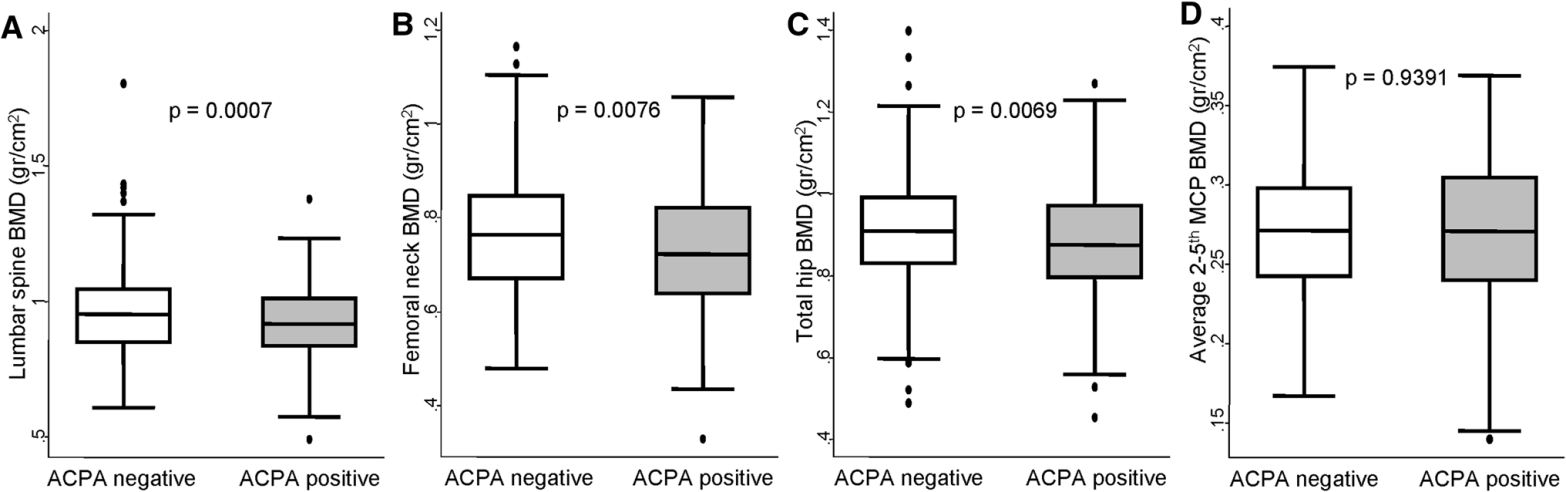
Systemic bone mineral density in anti-citrullinated protein antibodies (ACPA) positive patients. Panels represent distribution of bone mineral density (BMD) at different locations in patients included in this study: lumbar spine (**a**), femoral neck (**b**), total hip (**c**), and average of second to fifth metacarpophalangeal (MCP) joints (**d**). Data are presented as interquartile range (p75 *upper edge*, p25 *lower edge*, p50 *midline* in the box), p95 (*line* above the box) and p5 (*line* below the box). *Dots* represent outliers. Statistical significance was determined using the Mann–Whitney test and set at *p* < 0.0125 for multiple comparisons

ACPA-positive patients showed significantly lower unadjusted BMD at LS as well as at TH and FN (Fig. 1a–c). No significant differences were observed for the MCP joints (Fig. 1d). To determine whether differences in BMD between ACPA-positive and negative patients were a true effect of the autoantibodies or a bias related to differences in the characteristics of both populations, we fitted a multivariable analysis. In addition, we included in the model other variables that are known to have an influence on BMD, such as menopausal status, age, BMI, or cumulative glucocorticoid dose at the time of BMD measurement.

As expected, variability in BMD was significantly associated with gender, age, menopause, and BMI (Table 2). After adjustment for these variables, ACPA-positivity remained as an independent variable associated with lower values of BMD at LS, FN, and TH (Table 2). Disease duration at baseline, fulfillment of 2010 RA criteria, and disease activity, even when estimated with HUPI, that is more accurate than DAS28 [13], were excluded from the final models, since they did not significantly associate with differences in BMD at these locations and did not improve the models (data not shown).

**Table 2:** Effect of ACPA and other variables on bone mineral density (mg/cm^2^) at lumbar spine, hip, and MCP joints

	Lumbar spine (*n* = 553)		Femoral neck (*n* = 566)		Total hip (*n* = 566)		MCP 2nd–5th (*n* = 389)	
	*β* coef. (95% CI)	*p*	*β* coef. (95% CI)	*p*	*β* coef. (95% CI)	*p*	*β* coef. (95% CI)	*p*
ACPA-positive	−36 (−59; −12)	0.003	−23 (−41; − 5)	0.014	−25 (−50; −1)	0.046	2 (−6; 11)	0.572
Female	−32 (−64; 0)	0.053	−25 (−50; 0)	0.051	−64 (−98; −30)	< 0.001	−24 (−34; −13)	<0.001
Age (years)								
<45	Ref	–	Ref	–	Ref	–	Ref	–
45–65	−44 (−75; −13)	0.005	−54 (−79; −30)	<0.001	−49 (−82; −16)	0.003	−3 (−13; 7)	0.572
>65	−75 (−110; −40)	<0.001	−134 (−161; −107)	<0.001	−117 (−155; −81)	<0.001	−38 (−49; −27)	<0.001
BMI (kg/m^2^)	5 (3; 8)	<0.001	8 (6; 10)	<0.001	10 (8; 13)	<0.001	2 (2; 3)	<0.001
Menopause								
No	Ref	–	Ref	–	Ref	–	Ref	–
Yes	−69 (−99; −39)	<0.001	−53 (−77; −30)	<0.001	−50 (−81; −18)	0.002	−21 (−31; −12)	<0.001
Not available	−91 (−166; −17)	0.016	−35 (−94; 23)	0.238	−11 (−90; 69)	0.782	−25 (−46; −5)	0.014
2010 ACR/EULAR RA criteria	N.I		N.I		N.I		−8 (−17; 0)	0.050

*n* number, *coef*. coefficient, *CI* confidence interval, *ACPA* anti-citrullinated protein antibodies, *MCP* metacarpophalangeal joints, *BMI* body mass index, *RA* rheumatoid arthritis, *N.I*. not included

As at the other locations, variations in BMD at MCP joints were explained by gender, age, and BMI (Table 2). Furthermore, fulfillment of 2010 RA criteria was associated with a tendency toward lower BMD at MCP joints (Table 2). Surprisingly, the number of swollen MCP joints at the time of BMD measurement was not associated with lower bone mass at this location (data not shown).

A sensitivity analysis performed separately with the patients fulfilling 2010 RA criteria reproduced the findings described above (Supplementary data).

### MCV-ACPA-positivity is associated with lower systemic BMD

**Fig. 2:**
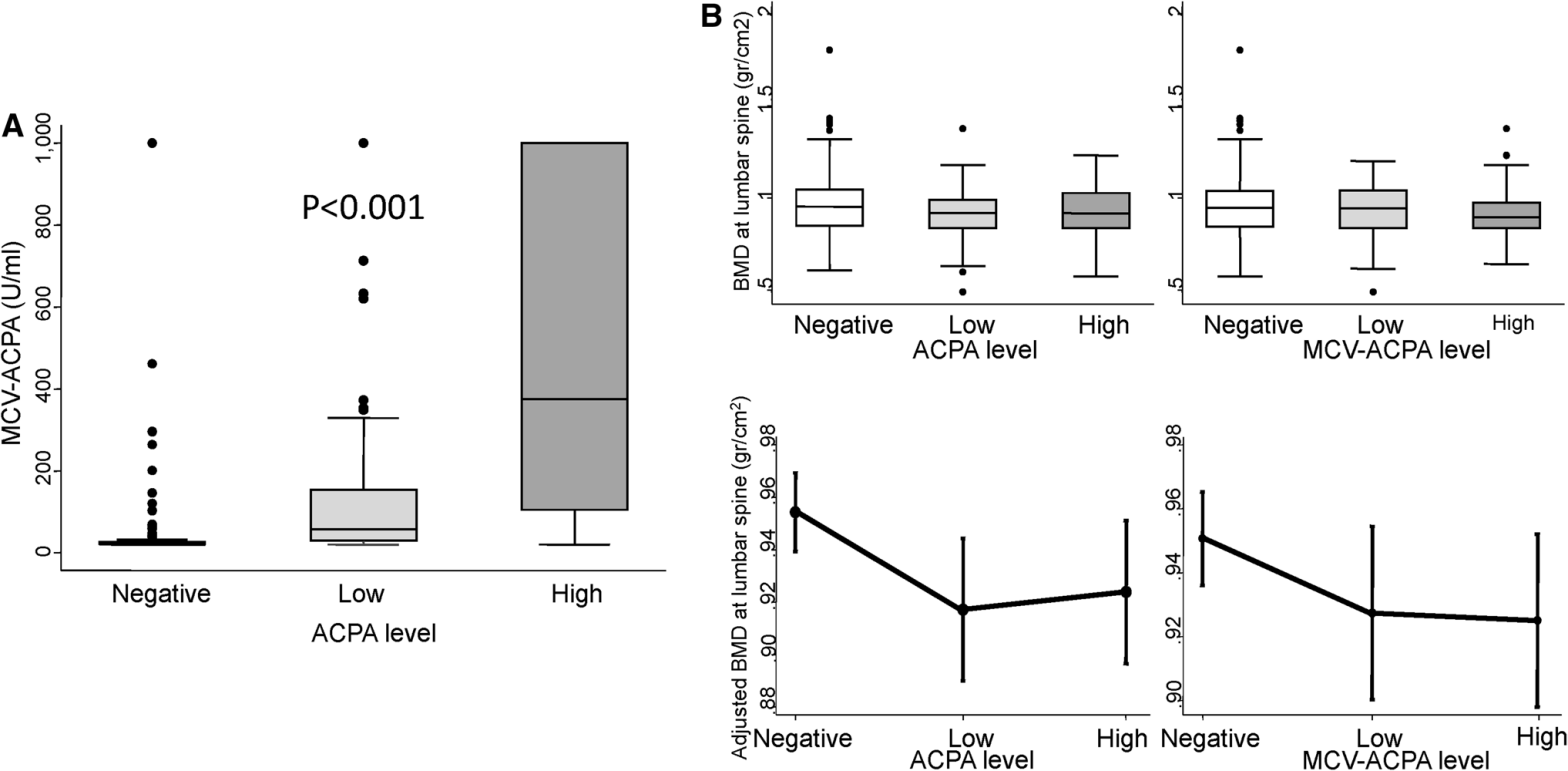
Correlation and comparative effect of anti-mutated citrullinated vimentin (MCV-ACPA) and anti-citrullinated proteins antibodies (ACPA) on lumbar spine bone mineral density. **a** Correlation between ACPA and MCV-ACPA levels. **b** Distribution of BMD at lumbar spine of patients from the Princesa Early Arthritis Register Longitudinal study according to ACPA levels (*left panels*) or MCV-ACPA levels (*right panels*). Data are presented as interquartile range (p75 *upper edge*, p25 *lower edge*, p50 *midline*), p95 (*line above the box*), and p5 (*line below the box*) in **a** and upper panels of **b**. *Dots* represent the outliers. Statistical significance was estimated using the Kruskal–Wallis test and was set at *p* < 0.05. In the lower panels of **b**, data are shown as the linear prediction with 95% confidence intervals for BMD at the lumbar spine according to the multivariable analysis (Tables 2, 3)

Finally, since MCV-ACPA have been associated with activation of osteoclasts and cortical osteoporosis in healthy individuals [9], we tested whether these antibodies were associated with lower BMD. The MCV-ACPA titers correlated significantly with ACPA levels (Fig. 2a). In addition, when we split our population according to MCV-ACPA status, BMD showed a pattern similar to that of ACPA (Fig. 2b). After adjustment for confounders, MCV-ACPA-positivity showed a similar tendency toward lower BMD at the same locations as total ACPA (Table 3).

**Table 3:** Effect of MCV-ACPA and other variables on bone mineral density (mg/cm^2^) at lumbar spine, hip, and MCP joints

	Lumbar spine (*n* = 523)		Femoral neck (*n* = 537)		Total hip (*n* = 537)		MCP 2nd–5th (*n* = 372)	
	*β* coef. (95% CI)	*p*	*β* coef. (95% CI)	*p*	*β* coef. (95% CI)	*p*	*β* coef. (95% CI)	*p*
MCV-ACPA level								
Negative	Ref	–	Ref	–	Ref	–	Ref	
Low	−27 (−58; 4)	0.086	−17 (−40; 7)	0.160	−30 (−54; −5)	0.018	7 (−4; 18)	0.218
High	−30 (−61; 1)	0.059	−9 (−33; 14)	0.447	−2 (−27; 22)	0.865	3 (−9; 13)	0.712
Female	−30 (−65; 6)	0.099	−23 (−50; 4)	0.092	−48 (−77; −20)	0.001	−24 (−35; −13)	<0.001
Age (years)								
<45	Ref	–	Ref	–	Ref	–	Ref	–
45–65	−45 (−77; −12)	0.007	−55 (−80; −30)	<0.001	−31 (−57; −5)	0.021	−5 (−15; 5)	0.331
>65	−68 (−106; −30)	<0.001	−134 (−163; −105)	<0.001	−105 (−135; −75)	<0.001	−40 (−51; −28)	<0.001
BMI (kg/m^2^)	5 (3; 8)	<0.001	9 (7; 11)	<0.001	11 (9; 13)	<0.001	2 (2; 3)	<0.001
Menopause								
No	Ref	–	Ref	–	Ref	–	Ref	–
Yes	−75 (−107; −43)	<0.001	−53 (−78; −28)	<0.001	−71 (−97; −45)	<0.001	−19 (−29; −9)	<0.001
Not available	−72 (−146; 1)	0.054	−31 (−86; 24)	0.268	−29 (−86; 29)	0.329	−22 (−41; −3)	0.023
2010 ACR/EULAR RA criteria	N.I		N.I		N.I		−9 (−19; 0)	0.055

*coef* coefficient, *CI* confidence interval, *MCV-ACPA* anti-mutated citrullinated vimentin antibodies, *ref*. reference, *BMI* body mass index, *RA* rheumatoid arthritis, *N.I*. not included, *MCP* metacarpophalangeal joints

## Discussion

Our results are important, since the previous studies that demonstrated this association were performed in vitro, in mice [5, 8] or in a small population of healthy individuals [9] where the influence of confounders could not be excluded. Recently, Bugatti et al. have published a study performed in a clinical setting demonstrating that the presence of ACPA and high RF levels is associated with lower systemic BMD, but not at juxta-articular bone level, in an early untreated RA population [17].

The higher prevalence of osteoporosis in RA patients than in the healthy general population remains common in the long-term disease [18]. The main explanations for this finding were thought to be the prolonged use of glucocorticoids and the persistent inflammatory activity during follow-up [19]. However, our previous data suggested that glucocorticoids may even have a beneficial effect on BMD when they are used over short periods to resolve inflammation in the early stages of the disease [20]. In that work, we analyzed the effect of disease activity and glucocorticoids in the variation of BMD in patients with RA after 2 years of follow-up. Cumulative disease activity was significantly associated with bone loss at lumbar spine and it showed a trend to significance at hip and ultradistal forearm, but no association was observed at mid forearm which is mainly cortical bone [20]. By contrast, 2 year cumulative dose of glucocorticoids was not associated with any significant effect on bone mass at hip, lumbar spine or hand [20]. In the present work, we did not observe any association between BMD and glucocorticoid use or disease activity. It is likely that the short disease duration avoids the detection of the deleterious effect of these variables on bone mass.

The presence of ACPA is thought to lead to local and systemic osteoporosis through osteoclasts activation even in the absence of chronic inflammation, although whether this effect is more intense in cortical [21] or trabecular [5] bone remains unclear. Our data support an inflammation-independent effect of ACPA based on the following reasons: (a) BMD measurements were performed early after the onset of arthritis; (b) The variation in BMD was not explained by the intensity of disease activity or disease duration. Nonetheless, this work does not clarify whether ACPA affect mainly cortical or trabecular bone, since the effect observed in our patients was most significant at the hip and LS, where bone mineral content is a mixture of cortical and trabecular bone.

Interestingly, Bugatti et al. found an association between the presence of ACPA and low BMD, defined as Z score ≤ −1 SD, at lumbar spine and total hip, an effect that was reinforced by the presence of high levels of RF [17].

Curiously, in our study, bone loss was more evidently associated with total ACPA than with MCV-ACPA. It may be related to technical issues in detection of MCV-ACPA compared with ACPA, leading to some discordance in their titers (Fig. 2a). However, the most likely explanation is that antibodies against citrullinated proteins other than vimentin have a similar effect on osteoclasts differentiation and activation. This is the case of anti-citrullinated enolase antibodies that have recently been associated with osteoclasts activation and bone loss in mice [5]. Other ACPA specificities, such as citrullinated fibrinogen or GRP78, induce monocyte or macrophage activation, leading altogether to the notion that ACPA have a pathogenic role in osteoporosis seen in patients with rheumatoid arthritis [22].

In addition, to autoimmunity-induced mechanisms, differences in bone microenvironment between anatomic sites cannot be ruled out. The ability of precursor cells to become mature osteoclasts may be affected by variations in the availability of osteoclasts precursors, the cytokine milieu, and cell–cell interactions [23]. Furthermore, heterogeneity in the phenotype of the resultant osteoclasts can also determine osteoclastogenic pathways with differences in bone resorption [23].

Our study has some limitations. Two different methods have been used to assess ACPA along the 14 years of the PEARL study. We think that considering ACPA as positive or negative or the semi-quantitative method used for normalization of their titers has minimized the impact of this issue. In addition, the method used during the last years was able to detect IgA and IgG ACPAs, whereas the first method only detected IgG. We do not know how this issue could affect our findings; nevertheless, osteoclast activation induced by ACPA has been described to be induced by complete ACPA but also by Fab ACPA, suggesting that this phenomenon is independent of the Fc fragment [8]. Therefore, it is likely that the use of two different methods to determine ACPA in our study had a little impact.

The heterogeneity of the population included could be considered a drawback of our study. However, similar findings were observed when we performed a sensitivity analysis separately in patients fulfilling or not the 2010 RA criteria. On the contrary, we consider that using a mixed population reinforces the effect of ACPA on BMD, since this variable proves to be significant in such a heterogeneous population independently of the clinical diagnosis.

Finally, we lack data on the age of menopause, a relevant variable for bone mineral density, in 2.3% of patients included in the study. There were no significant differences in the percentage of patients with menopause and those with no available information on menopause status between ACPA-positive or ACPA-negative patients, so we consider that this issue does not affect significantly our findings of association between low bone mass and ACPA-positivity.

In conclusion, our data support the previous observations, suggesting that ACPA are associated with bone loss in patients submitted by suspicion of arthritis, independently of the etiology. Further studies are necessary to determine their clinical relevance, since, although the effect of ACPA on BMD was significant, the long-term clinical impact of these findings is currently unknown. Studies exploring whether there are differences in the prevalence of osteoporotic fractures between ACPA-positive and negative patients would be needed to determine the real impact of these findings.

## Electronic supplementary material

Below is the link to the electronic supplementary material.

Supplementary material 1 (DOCX 18 KB)

## References

[1] Goldring SR, Gravallese EM (2000). Mechanisms of bone loss in inflammatory arthritis: diagnosis and therapeutic implications. Arthritis Res.

[2] Schett G, Gravallese E (2012). Bone erosion in rheumatoid arthritis: mechanisms, diagnosis and treatment. Nat Rev Rheumatol.

[3] Kocijan R, Harre U, Schett G (2013). ACPA and bone loss in rheumatoid arthritis. Curr Rheumatol Rep.

[4] Schett G, Teitelbaum SL (2009). Osteoclasts and Arthritis. J Bone Min Res.

[5] Krishnamurthy A, Joshua V, Haj Hensvold A (2016). Identification of a novel chemokine-dependent molecular mechanism underlying rheumatoid arthritis-associated autoantibody-mediated bone loss. Ann Rheum Dis.

[6] Walsh NC, Crotti TN, Goldring SR, Gravallese EM (2005). Rheumatic diseases: the effects of inflammation on bone. Immunol Rev.

[7] Boyesen P, Hoff M, Odegard S (2009). Antibodies to cyclic citrullinated protein and erythrocyte sedimentation rate predict hand bone loss in patients with rheumatoid arthritis of short duration: a longitudinal study. Arthritis Res Ther.

[8] Harre U, Georgess D, Bang H (2012). Induction of osteoclastogenesis and bone loss by human autoantibodies against citrullinated vimentin. J Clin Invest.

[9] Kleyer A, Finzel S, Rech J (2014). Bone loss before the clinical onset of rheumatoid arthritis in subjects with anticitrullinated protein antibodies. Ann Rheum Dis.

[10] Esteve-Vives J, Batlle-Gualda E, Reig A (1993). Spanish version of the Health Assessment Questionnaire: reliability, validity and transcultural equivalency. Grupo para la Adaptacion del HAQ a la Poblacion Espanola. J Rheumatol.

[11] Prevoo ML, van ‘t Hof MA, Kuper HH, van Leeuwen MA, van de Putte LB, van Riel PL (1995). Modified disease activity scores that include twenty-eight-joint counts. Development and validation in a prospective longitudinal study of patients with rheumatoid arthritis. Arthritis Rheum.

[12] Castrejon I, Carmona L, Ortiz AM, Belmonte MA, Martinez-Lopez JA, Gonzalez-Alvaro I (2013). Development and validation of a new disease activity index as a numerical sum of four variables in patients with early arthritis. Arthritis Care Res (Hoboken).

[13] Gonzalez-Alvaro I, Castrejon I, Ortiz AM (2016). Cut-offs and response criteria for the hospital Universitario La Princesa Index (HUPI) and their comparison to widely-used indices of disease activity in rheumatoid arthritis. PLoS One.

[14] Gonzalez-Alvaro I, Ortiz AM, Alvaro-Gracia JM (2011). Interleukin 15 levels in serum may predict a severe disease course in patients with early arthritis. PLoS One.

[15] Castaneda S, Gonzalez-Alvaro I, Rodriguez-Salvanes F, Quintana ML, Laffon A, Garcia-Vadillo JA (2007). Reproducibility of metacarpophalangeal bone mass measurements obtained by dual-energy X-ray absorptiometry in healthy volunteers and patients with early arthritis. J Clin Densitom.

[16] Felson DT, Smolen JS, Wells G (2011). American College of Rheumatology/European League Against Rheumatism provisional definition of remission in rheumatoid arthritis for clinical trials. Arthritis Rheum.

[17] Bugatti S, Bogliolo L, Vitolo B, Manzo A, Montecucco C, Caporali R (2016). Anti-citrullinated protein antibodies and high levels of rheumatoid factor are associated with systemic bone loss in patients with early untreated rheumatoid arthritis. Arthritis Res Ther.

[18] Haugeberg G, Uhlig T, Falch JA, Halse JI, Kvien TK (2000). Bone mineral density and frequency of osteoporosis in female patients with rheumatoid arthritis: results from 394 patients in the Oslo County Rheumatoid Arthritis register. Arthritis Rheum.

[19] Solomon DH, Kuntz KM (2000). Should postmenopausal women with rheumatoid arthritis who are starting corticosteroid treatment be screened for osteoporosis? A cost-effectiveness analysis. Arthritis Rheum.

[20] Ibanez M, Ortiz AM, Castrejon I (2010). A rational use of glucocorticoids in patients with early arthritis has a minimal impact on bone mass. Arthritis Res Ther.

[21] Kleyer A, Schett G (2014). Arthritis and bone loss: a hen and egg story. Curr Opin Rheumatol.

[22] Sokolove J, Pisetsky D (2016). Bone loss, pain and inflammation: three faces of ACPA in RA pathogenesis. Ann Rheum Dis.

[23] Adamopoulos IE, Mellins ED (2015). Alternative pathways of osteoclastogenesis in inflammatory arthritis. Nat Rev Rheumatol.

