# Supplementary material for: Anti-citrullinated protein antibodies are associated with decreased bone mineral density: baseline data from a register of early arthritis patients
Source: Rheumatol Int. 2017 Feb 27;37(5):799–806. doi: 10.1007/s00296-017-3674-9 (PMC5397447; doi:10.1007/s00296-017-3674-9)
Supplement: Supplementary file 1 — Supplementary material 1 (DOCX 18 KB) [file 296_2017_3674_MOESM1_ESM.docx]

**Supplementary Table 1.** Multivariable analysis. Variables associated with bone mineral density (mg/cm^2^) at different locations (lumbar spine, hip and MCP). Sensitivity analysis including only patients fulfilling 2010 RA criteria.

|  | **Lumbar spine (n=298)** | | **Femoral neck (n=310)** | | **Total hip (n=310)** | | **MCP 2^nd^-5^th^ (n=228)** | |
| --- | --- | --- | --- | --- | --- | --- | --- | --- |
|  | 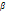β coef.(95%CI) | p | 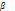β coef.(95%CI) | p | 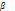β coef.(95%CI) | p | 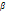β coef.(95%CI) | p |
| **ACPA-positive** | -40 (-70; -10) | 0.009 | -21 (-45; 3) | 0.083 | -24 (-62; 15) | 0.229 | 3 (-7; 14) | 0.529 |
| **Female** | -56 (-99; -12) | 0.012 | -50 (-85; - 15) | 0.005 | -104 (-160; -48) | <0.001 | -28 (-43; -13) | <0.001 |
| **Age (years)** |  |  |  |  |  |  |  |  |
| **<45** | Ref | - | Ref. | - | Ref. | - | Ref. | - |
| **45-65** | -80 (-116; -43) | <0.001 | -83 (-115; -50) | <0.001 | -93 (-145; -41) | <0.001 | -10 (-24; 4) | 0.155 |
| **>65** | -154 (-196; -113) | <0.001 | -169 (-206; -132) | <0.001 | -165 (-224; -106) | <0.001 | -51 (-67; -36) | <0.001 |
| **BMI (kg/m^2^)** | 5 (2; 7) | 0.003 | 9 (7; 12) | <0.001 | 10 (6; 14) | <0.001 | 2 (1; 3) | <0.001 |
| **Menopause** |  |  |  |  |  |  |  |  |
| **No** | Ref | - | Ref | - | Ref | - | Ref | - |
| **Yes** | -43 (-80; -6) | 0.023 | -29 (-59; 2) | 0.064 | -16 (-64; 33) | 0.524 | -13 (-26; -1) | 0.036 |
| **Not available** | -66 (-147; 14) | 0.107 | 1 (-65; 67) | 0.982 | 29 (-76; 134) | 0.589 | -21 (-46; 4) | 0.105 |

**Abbreviations**: coef.: coefficient; CI: confidence interval; ACPA: anti-citrullinated protein antibodies; ref.: reference; BMI: body mass index; MCP: metacarpophalangeal joints.
